# Posterior segment imaging clues to systemic malignancy: a phenotype-to-confirmation narrative review

**DOI:** 10.3389/fopht.2026.1917738

**Published:** 2026-09-28

**Authors:** Nikola Denda, Sofija Davidović Terzić, Nemanja Maletin, Kristina Veljkov, Aleksandar Rasković, Ljiljana Jovančević, Siniša Babović

**Affiliations:** 1 Eye Clinic, University Clinical Center of Vojvodina, Novi Sad, Serbia; 2 Faculty of Medicine, University of Novi Sad, Novi Sad, Serbia; 3 Clinic for Otorhinolaryngology and Head and Neck Surgery, University Clinical Center of Vojvodina, Novi Sad, Serbia

**Keywords:** choroidal metastasis, multimodal imaging, posterior segment, systemic malignancy, vitreoretinal lymphoma

## Abstract

Systemic malignancies may affect the posterior segment through metastatic, paraneoplastic, hematologic or lymphomatous, and treatment-related mechanisms. Because routine ophthalmic imaging rarely establishes the underlying etiology, its principal clinical value is to identify suspicious patterns, prioritize competing diagnoses, and guide further investigation. This narrative review synthesizes malignancy-related posterior segment findings and proposes a clinically oriented phenotype-to-confirmation framework for multimodal interpretation. A literature search was conducted in PubMed, Scopus, and Web of Science for publications from January 2000 to May 18, 2026. The review included choroidal metastasis, paraneoplastic retinopathies and choroidopathies, vitreoretinal lymphoma, hematologic infiltration, leukemic retinopathy, and anticancer therapy-related toxicity. Owing to heterogeneity in disease entities, imaging protocols, diagnostic confirmation, and study designs, the evidence was synthesized qualitatively. Findings were organized into five suspicion-triggering phenotypes: exudative choroidal mass, RPE-choroidal remodeling, vitreoretinal infiltrative, diffuse neuroretinal dysfunction, and treatment-imprint phenotypes. Each phenotype was linked to its principal mimickers and to appropriate additional investigations, including targeted multimodal imaging, electrophysiology, ocular-fluid analysis, molecular testing, or systemic oncologic reassessment. These phenotypes should not be regarded as validated diagnostic categories, but as clinical entry points for differential exclusion and diagnostic escalation. Artificial intelligence may eventually support image registration, lesion tracking, and longitudinal comparison; however, its role in these rare and heterogeneous disorders remains investigational.

## 1 Introduction

Cancer remains a leading cause of morbidity and mortality worldwide. Systemic malignancies may affect the posterior segment through direct metastatic spread, hematologic or lymphomatous infiltration, paraneoplastic immune-mediated mechanisms, and adverse effects of anticancer therapy (1). The retina, retinal pigment epithelium (RPE), and choroid are vascularly and metabolically specialized tissues whose structural abnormalities can be documented non-invasively and followed longitudinally (2). Posterior segment findings may therefore provide clinically relevant clues to an underlying malignancy, disease recurrence, systemic treatment effect, or an alternative ocular disorder requiring exclusion. Direct metastatic involvement may produce focal or multifocal choroidal masses with secondary subretinal fluid and RPE disturbance. By contrast, paraneoplastic entities such as cancer-associated retinopathy, melanoma-associated retinopathy, bilateral diffuse uveal melanocytic proliferation, and paraneoplastic vitelliform-like maculopathy can cause retinal and choroidal dysfunction without direct tumor invasion (3–6). Malignancy-related posterior segment disease may present with visual loss, photopsia, nyctalopia, metamorphopsia, subretinal fluid, RPE alteration, outer-retinal disruption, choroidal thickening, vitritis, or abnormal autofluorescence patterns (7). Multimodal imaging, including color or ultra-widefield fundus photography, optical coherence tomography, enhanced-depth or swept-source OCT, fundus autofluorescence, fluorescein and indocyanine green angiography, OCT angiography, and ocular ultrasonography, has improved the detection, anatomic localization, and longitudinal characterization of these abnormalities (8, 9). However, the same elementary findings may occur in inflammatory, infectious, vascular, degenerative, or treatment-related disorders. Imaging can therefore increase or decrease clinical suspicion, but rarely establishes the underlying etiology independently of the clinical context and appropriate confirmatory investigation (10).

The specific contribution of this narrative review is the integration of metastatic, paraneoplastic, hematologic or lymphomatous, and treatment-related posterior segment abnormalities into a shared phenotype-to-confirmation framework. Rather than providing an exhaustive disease-centered catalogue, the review is organized around a practical clinical question: when should an atypical posterior segment pattern raise concern for a malignancy-related mechanism, which competing diagnoses should be prioritized, and which additional ocular, molecular, laboratory, or systemic investigation should follow? The proposed phenotypes are therefore presented as suspicion-triggering clinical entry points rather than validated diagnostic categories. Their intended role is to support differential exclusion, selection of the most appropriate next diagnostic step, and timely multidisciplinary communication between ophthalmologists, oncologists, hematologists, and other relevant specialists.

## 2 Methods

### 2.1 Review design and scope

This study was conducted as a structured narrative review with a targeted literature search focused on retinal, retinal pigment epithelial (RPE), choroidal, vitreous, and retinal vascular findings associated with systemic malignancy. A narrative design was selected because the topic encompasses heterogeneous metastatic, paraneoplastic, hematologic, lymphomatous, infiltrative, and treatment-related posterior segment entities that differ substantially in prevalence, diagnostic confirmation, imaging protocols, follow-up duration, and level of available evidence.

The aim was not to perform a systematic review, meta-analysis, or diagnostic accuracy assessment, but to develop a clinically oriented phenotype-to-confirmation framework for multimodal interpretation in onco-ophthalmology. The review therefore focused on posterior segment abnormalities involving the retina, RPE, choroid, vitreous, subretinal and sub-RPE spaces, and retinal vasculature in patients with known, suspected, recurrent, or occult systemic malignancy. Primary uveal and retinal tumors were not a principal focus and were considered primarily when relevant to the differential diagnosis of malignancy-related posterior segment findings. Vitreoretinal lymphoma was included because of its close association with primary central nervous system lymphoma and its clinically important role as a masquerade syndrome. Within the proposed framework, an imaging phenotype is not considered a definitive diagnosis. Instead, it is used to raise or lower clinical suspicion, prioritize relevant infectious, inflammatory, degenerative, and neoplastic mimickers, and identify the most appropriate next diagnostic step, including additional imaging, ocular fluid or tissue sampling, molecular testing, systemic oncologic reassessment, or treatment-toxicity evaluation.

### 2.2 Literature search strategy

A targeted literature search was performed in PubMed, Scopus, and Web of Science for articles published from January 2000 to May 18, 2026. The search strategy combined four concept groups: malignancy-related disease entities, posterior segment anatomical structures, imaging and functional assessment modalities, and methods of diagnostic confirmation. Disease-related terms included *choroidal metastasis*, *retinal metastasis*, *cancer-associated retinopathy*, *melanoma-associated retinopathy*, *autoimmune retinopathy*, *paraneoplastic retinopathy*, *paraneoplastic choroidopathy*, *bilateral diffuse uveal melanocytic proliferation*, *paraneoplastic vitelliform maculopathy*, *vitreoretinal lymphoma*, *leukemic retinopathy*, *hematologic infiltration*, *ocular metastasis*, and *posterior segment malignancy*. Anatomical terms included *retina*, *choroid*, *retinal pigment epithelium*, *RPE*, *vitreous*, *subretinal*, *sub-RPE*, and *retinal vasculature*. Imaging and functional assessment terms included *optical coherence tomography*, *OCT*, *enhanced-depth imaging OCT*, *EDI-OCT*, *swept-source OCT*, *fundus autofluorescence*, *FAF*, *fluorescein angiography*, *FA*, *indocyanine green angiography*, *ICGA*, *OCT angiography*, *OCTA*, *B-scan ultrasonography*, *widefield imaging*, *ultra-widefield imaging*, *multimodal imaging*, *electroretinography*, *ERG*, and *imaging biomarker*. Diagnostic-confirmation terms included *biopsy*, *vitreous sampling*, *aqueous sampling*, *cytology*, *flow cytometry*, *immunophenotyping*, *interleukin-10*, *interleukin-6*, *MYD88*, *immunoglobulin gene rearrangement*, *clonality*, *polymerase chain reaction*, *next-generation sequencing*, and *molecular diagnosis*. Search syntax and combinations were adapted to the indexing structure of each database. Although the primary database search was restricted to publications from 2000 onward, selected seminal publications published before 2000 were retained when they were identified through backward citation searching and had established a canonical clinical or imaging phenotype relevant to the review. Reference lists of relevant reviews, clinical studies, case series, consensus statements, and key articles were screened manually to identify additional publications, particularly for rare entities and reports containing detailed multimodal imaging or diagnostic-confirmation data.

The literature search was conducted iteratively across the predefined disease groups rather than as a single pooled systematic query. During the final eligibility reassessment, 55 candidate publications identified through targeted database searches and backward citation screening were evaluated for relevance to the review objectives. Three publications were excluded because their relevance to systemic malignancy-related posterior segment disease was indirect or because they provided redundant background evidence, particularly in the AI section. A total of 52 publications were retained for the final qualitative synthesis.

Of the 52 included publications, 28 primarily informed the five phenotype-specific clinical and imaging domains: exudative choroidal mass (n = 9), RPE–choroidal remodeling (n = 6), vitreoretinal infiltrative (n = 5), diffuse neuroretinal dysfunction (n = 5), and treatment-imprint phenotypes (n = 3). Eleven publications provided supporting evidence on general multimodal imaging principles, ocular oncology, and differential diagnosis, while thirteen addressed AI-assisted interpretation or longitudinal digital monitoring. Because some publications contributed to more than one topic, each source was assigned to the category representing its principal contribution to the qualitative synthesis.

### 2.3 Eligibility criteria and article selection

Publications were considered eligible if they addressed posterior segment manifestations associated with systemic malignancy and provided clinically relevant information on multimodal imaging, differential diagnosis, diagnostic confirmation, disease evolution, treatment-related ocular findings, or ophthalmology–oncology decision-making. Eligible publication types included clinical studies, observational cohorts, retrospective series, case series, selected case reports, diagnostic imaging studies, consensus statements, and relevant narrative or systematic reviews. Case reports were included selectively when they provided detailed multimodal imaging documentation together with histologic, cytologic, molecular, systemic oncologic, or treatment-response correlation, or when they contributed clinically important diagnostic insight into a rare presentation. When available, greater interpretive weight was given to publications in which the diagnosis was supported by histopathology, cytology, molecular testing, systemic oncologic evaluation, or a well-documented longitudinal clinical course.

Articles were excluded if they focused exclusively on primary intraocular tumors without relevance to the differential diagnosis of systemic malignancy-related findings, isolated anterior segment abnormalities, orbital disease without posterior segment involvement, or ocular findings described only incidentally without sufficient clinical, imaging, or diagnostic detail. Titles and abstracts were screened initially, followed by full-text assessment of potentially relevant publications.

Final inclusion was guided by clinical relevance, quality of imaging description, strength of diagnostic or systemic oncologic correlation, availability of longitudinal information, and contribution to the phenotype-to-confirmation framework. Because this was a narrative review rather than a systematic evidence synthesis, PRISMA-based reporting, formal risk-of-bias assessment, quantitative evidence grading, and pooled diagnostic accuracy estimates were not applied. Accordingly, the proposed phenotypes should be interpreted as clinically oriented conceptual categories rather than as evidence-graded diagnostic criteria.

### 2.4 Qualitative synthesis and phenotype development

Findings were synthesized qualitatively by identifying recurring multimodal patterns across metastatic, paraneoplastic, hematologic, infiltrative, lymphomatous, and treatment-related posterior segment disease. The synthesis considered five interpretive dimensions: the dominant anatomical compartment, lesion morphology, presumed oncologic mechanism, principal competing diagnoses, and potential clinical actionability. Dominant anatomical compartments included the choroid, RPE, sub-RPE space, neurosensory retina, vitreous, and retinal vasculature. Lesion morphology was categorized as mass-like, exudative, infiltrative, inflammatory, ischemic, degenerative, or toxic. Presumed mechanisms included hematogenous metastatic spread, paraneoplastic immune-mediated injury, hematologic or lymphomatous infiltration, and anticancer therapy-related toxicity. Competing diagnoses included infectious, inflammatory, vascular, degenerative, and primary ocular neoplastic disorders that may produce overlapping imaging findings. Clinical actionability was evaluated according to whether a pattern could help prioritize the differential diagnosis, indicate the need for an additional imaging modality, prompt ocular fluid or tissue sampling, guide microbiological or molecular testing, trigger oncologic or hematologic reassessment, identify possible treatment-related toxicity, or support longitudinal monitoring.

Through this iterative qualitative synthesis, malignancy-related findings were organized into five proposed morphologic imaging phenotypes: the exudative choroidal mass phenotype, RPE–choroidal remodeling phenotype, vitreoretinal infiltrative phenotype, diffuse neuroretinal dysfunction phenotype, and treatment-imprint phenotype. These categories were developed as a practical interpretive model and should be regarded as suspicion-triggering imaging patterns linked to differential diagnoses and next-step investigations, rather than as validated diagnostic entities, scoring systems, or independent predictors of malignancy. The narrative search and synthesis approach is summarized in **Table 1**.

**Table 1:** Narrative search and synthesis approach.

Domain	Description
Review type	Structured narrative review with a targeted literature search
Databases	PubMed, Scopus, and Web of Science
Search period	January 2000 to May 18, 2026, with selected earlier seminal publications identified through backward citation searching
Main disease groups	Choroidal and retinal metastases, paraneoplastic retinopathies and choroidopathies, vitreoretinal lymphoma, hematologic infiltration, leukemic retinopathy, and anticancer therapy-related toxicity
Anatomical scope	Retina, RPE, choroid, vitreous, subretinal and sub-RPE spaces, and retinal vasculature
Imaging and functional assessment	OCT, EDI-OCT, swept-source OCT, FAF, FA, ICGA, OCTA, B-scan ultrasonography, widefield and ultra-widefield imaging, and ERG
Diagnostic-confirmation focus	Ocular fluid or tissue sampling, cytology, immunophenotyping or flow cytometry, cytokine analysis, pathogen-specific PCR, clonality assessment, MYD88 testing, and targeted next-generation sequencing
Included evidence	Clinical studies, observational cohorts, retrospective series, case series, selected diagnostically informative case reports, diagnostic imaging studies, consensus statements, and relevant reviews
Excluded evidence	Primary intraocular tumors without relevance to systemic malignancy-related differential diagnosis, anterior segment-only findings, orbital disease without posterior segment involvement, and reports with insufficient clinical or imaging detail
Synthesis method	Qualitative synthesis according to anatomical compartment, morphology, mechanism, competing diagnoses, and clinical actionability
Output	Five proposed suspicion-triggering imaging phenotypes linked to differential diagnoses and next-step investigations

EDI-OCT, enhanced-depth imaging optical coherence tomography; ERG, electroretinography; FA, fluorescein angiography; FAF, fundus autofluorescence; ICGA, indocyanine green angiography; OCT, optical coherence tomography; OCTA, optical coherence tomography angiography; PCR, polymerase chain reaction; RPE, retinal pigment epithelium.

## 3 Results

### 3.1 Overview and definition of suspicion-triggering phenotypes

In this review, the term suspicion-triggering imaging pattern is used in a broad clinical and imaging-based sense to describe retinal, retinal pigment epithelial (RPE), and choroidal findings that may indicate the presence, recurrence, progression, or treatment-related consequences of systemic malignancy. The term does not imply validated diagnostic accuracy, independent predictive value, or a definitive image-based diagnosis. Rather, it refers to an ocular pattern whose oncologic relevance depends on integration with patient age, systemic cancer history, treatment exposure, laterality, disease tempo, and longitudinal evolution. The principal clinical value of these patterns is therefore to raise or lower suspicion, prioritize competing diagnoses, and direct the clinician toward appropriate additional ocular, laboratory, molecular, or systemic evaluation (11, 12).

Malignancy-related retinal and choroidal abnormalities can be broadly organized into four mechanistic categories: direct metastatic involvement, paraneoplastic retinal or choroidal syndromes, hematologic or lymphomatous infiltration, and treatment-related posterior segment changes (13). Each category reflects a different mechanism of ocular involvement and consequently requires a different diagnostic pathway. Direct metastatic disease most often produces a focal or multifocal choroidal lesion with secondary subretinal fluid and RPE disturbance. By contrast, paraneoplastic disease may cause diffuse retinal dysfunction, RPE alteration, choroidal remodeling, or characteristic autofluorescence abnormalities without direct tumor invasion (14).

The choroid is particularly relevant in systemic malignancy because of its rich vascular supply and susceptibility to hematogenous tumor dissemination. The retina and RPE may instead be affected through immune-mediated, infiltrative, toxic, vascular, or treatment-related mechanisms (15). Accordingly, posterior segment abnormalities should not be interpreted as isolated imaging signs, but as components of a broader metastatic, paraneoplastic, infiltrative, or therapy-related clinical pattern.

Practical recognition depends on pattern-based interpretation. Combinations of lesion morphology, anatomic localization, distribution, laterality, treatment exposure, and longitudinal behavior may support a malignancy-related mechanism over a common retinal or inflammatory disorder (16, 17). However, no individual imaging feature is pathognomonic. Imaging findings must be interpreted together with systemic symptoms, known or suspected cancer, previous treatment, response to ophthalmic therapy, and evolution over time (18, 19).

Within this framework, the mechanistic categories provide the pathophysiologic basis, whereas the proposed phenotypes function as clinical entry points for differential diagnosis and diagnostic escalation.

### 3.2 Phenotype-specific imaging clues and differential exclusion

Building on this approach, malignancy-related posterior segment abnormalities can be organized into five proposed morphologic phenotypes. These phenotypes are not intended to replace disease-specific diagnoses or to function as validated scoring systems. Their purpose is to connect the dominant ocular pattern with a plausible mechanism, define the most relevant mimickers, and identify when additional investigation is warranted (20). The principal features are summarized in **Table 2**.

**Table 2:** Proposed suspicion-triggering phenotypes of systemic malignancy-related posterior segment disease.

Suspicion-triggering phenotype	Dominant imaging pattern	Principal malignancy-associated interpretation	Priority differential diagnoses	Clinical implication
Exudative choroidal mass	Amelanotic or creamy-yellow choroidal lesion, subretinal fluid, RPE disturbance, altered choroidal contour on OCT	Choroidal metastasis	Amelanotic melanoma, choroidal hemangioma, inflammatory granuloma, posterior scleritis	A new, progressive, multifocal, or otherwise unexplained lesion may warrant ocular-oncology assessment and systemic reassessment
RPE-choroidal remodeling	Multifocal RPE change, abnormal FAF, subretinal fluid, choroidal thickening, bilateral or diffuse involvement	BDUMP or another paraneoplastic choroidopathy	Central serous chorioretinopathy, VKH disease, posterior scleritis, inflammatory choroidopathy, drug-related change	Bilateral, progressive, or atypical RPE-choroidal abnormalities may warrant malignancy-directed systemic evaluation after common mimickers have been assessed
Vitreoretinal infiltrative	Persistent or recurrent vitritis, vitreous cellular aggregates, yellow-white retinal or subretinal lesions, sub-RPE infiltrates, transient or incomplete corticosteroid response	Vitreoretinal lymphoma or hematologic infiltration	Infectious retinitis, intermediate or posterior uveitis, sarcoidosis, syphilis, tuberculosis, white-dot syndromes	An atypical, recurrent, or treatment-resistant inflammatory presentation should trigger a structured masquerade-syndrome work-up
Diffuse neuroretinal dysfunction	Photopsia, nyctalopia, visual-field loss, outer-retinal attenuation, ellipsoid-zone disruption, abnormal ERG	Cancer-associated retinopathy, melanoma-associated retinopathy, or another paraneoplastic autoimmune retinopathy	Inherited retinal degeneration, toxic retinopathy, AZOOR spectrum, non-paraneoplastic autoimmune retinopathy	Functional impairment disproportionate to fundus findings should prompt electrophysiologic assessment and systemic correlation
Treatment-imprint	Serous retinal change, macular edema, inflammatory uveoretinal disease, vascular events, radiation retinopathy, toxic outer-retinal change	Anticancer therapy-related toxicity or immune-mediated ocular adverse event	Tumor progression, opportunistic infection, diabetic or hypertensive retinopathy, retinal vascular occlusion, unrelated inflammatory disease or drug toxicity	A plausible temporal relationship should prompt toxicity assessment and multidisciplinary ophthalmology-oncology discussion before systemic treatment is modified

AZOOR, acute zonal occult outer retinopathy; BDUMP, bilateral diffuse uveal melanocytic proliferation; ERG, electroretinography; FAF, fundus autofluorescence; OCT, optical coherence tomography; RPE, retinal pigment epithelium; VKH, Vogt-Koyanagi-Harada disease.

The exudative choroidal mass phenotype is suggested by an amelanotic or creamy-yellow choroidal lesion associated with subretinal fluid, RPE disturbance, and an altered choroidal contour on OCT (21). This combination should raise suspicion for choroidal metastasis, particularly in an older patient, a patient with known systemic malignancy, or a patient with new systemic symptoms. Nevertheless, amelanotic melanoma, choroidal hemangioma, inflammatory granuloma, and posterior scleritis remain important alternatives (22).

The RPE-choroidal remodeling phenotype comprises multifocal RPE alterations, abnormal fundus autofluorescence (FAF), subretinal fluid, choroidal thickening, and bilateral or diffuse involvement (23). This pattern may occur in paraneoplastic choroidopathies, particularly bilateral diffuse uveal melanocytic proliferation (BDUMP), but overlaps substantially with central serous chorioretinopathy, Vogt-Koyanagi-Harada (VKH) disease, posterior scleritis, inflammatory choroidopathy, and drug-related change (6, 24).

The vitreoretinal infiltrative phenotype is suggested by persistent or recurrent vitritis, large vitreous cells or cellular aggregates, yellow-white retinal or subretinal lesions, sub-RPE infiltrates, and a transient or incomplete response to corticosteroids (25). In an older patient, or in recurrent, atypical, or treatment-resistant inflammation, this pattern should prompt consideration of vitreoretinal lymphoma or hematologic infiltration. It remains a suspicion-triggering pattern rather than a diagnostic definition because infectious and non-infectious uveitic disorders may produce overlapping findings.

The diffuse neuroretinal dysfunction phenotype is dominated by functional symptoms and tests, including photopsia, nyctalopia, visual-field loss, outer-retinal attenuation, ellipsoid-zone disruption, and abnormal electroretinography (ERG) (26). This pattern is compatible with paraneoplastic autoimmune retinopathy, including cancer-associated and melanoma-associated retinopathy, but overlaps with inherited retinal degeneration, toxic retinopathy, the acute zonal occult outer retinopathy (AZOOR) spectrum, and non-paraneoplastic autoimmune retinopathy (6, 27).

The treatment-imprint phenotype comprises posterior segment abnormalities temporally associated with systemic anticancer therapy (28). Depending on the treatment class, these may include serous retinal changes, macular edema, inflammatory uveoretinal disease, vascular events, radiation retinopathy, or toxic outer-retinal alterations. Interpretation requires correlation with the type, timing, duration, and sequence of anticancer therapy and careful exclusion of tumor progression, opportunistic infection, and unrelated retinal disease.

Overall, the proposed phenotype-based framework provides a bridge between multimodal imaging and oncologic reasoning. Its purpose is not to label an abnormality as malignant, but to determine whether the observed pattern can be adequately explained by a common ocular disorder or whether malignancy-directed investigation is justified.

### 3.3 From a suspicious phenotype to differential diagnosis and confirmatory work-up

Once a dominant phenotype has been recognized, the next task is not to assign a definitive diagnosis from imaging alone, but to determine which competing mechanisms require exclusion and which additional investigation is most likely to resolve the uncertainty. A practical assessment should first localize the dominant ocular compartment; characterize the process as mass-like, exudative, infiltrative, immune-mediated, ischemic, degenerative, or treatment-related; assess lesion distribution, laterality, and change over time; and integrate the ocular findings with systemic cancer history, symptoms, treatment exposure, immune status, and response to previous therapy (5, 29).

Because individual findings may overlap between phenotypes, diagnostic escalation should be guided by the overall combination of clinical and multimodal features rather than by any isolated sign.

#### 3.3.1 Exudative choroidal mass phenotype

In a patient with a choroidal mass, the diagnostic objective is to determine whether the lesion behaves as an exudative malignant deposit or as a melanocytic, vascular, inflammatory, or benign tumor-like process (30). Suspicion for choroidal metastasis increases when a creamy-yellow or amelanotic lesion is accompanied by subretinal fluid, secondary RPE disturbance, documented rapid enlargement, multifocality, or bilateral involvement. Although none of these features is pathognomonic, their combination is particularly relevant in an older patient, a patient with known malignancy, or a patient with new systemic symptoms.

EDI-OCT or swept-source OCT may demonstrate a plateau-shaped or lobulated anterior choroidal contour, compression or disruption of the overlying choriocapillaris and RPE, and associated subretinal fluid. A markedly irregular or “lumpy-bumpy” surface may support suspicion for metastasis but should not be interpreted as histologically specific. FAF frequently demonstrates secondary hyperautofluorescent or hypoautofluorescent changes related to subretinal fluid, lipofuscin accumulation, or RPE damage; these findings reflect the response of the overlying RPE rather than the tumor type itself. Widefield imaging is useful for documenting the full lesion extent and detecting multifocal or peripheral deposits that may be underestimated on conventional examination.

Standardized A- and B-scan ultrasonography should document lesion thickness, basal dimensions, configuration, internal reflectivity, and associated retinal or choroidal detachment. Choroidal metastases often demonstrate a flatter or dome-shaped configuration and medium-to-high internal reflectivity, whereas an amelanotic melanoma may be more commonly associated with lower internal reflectivity, intrinsic vascularity, or a dome-shaped or mushroom configuration. These acoustic tendencies are not absolute. Choroidal hemangioma may demonstrate high internal reflectivity and characteristic vascular filling, while posterior scleritis should be considered when pain, diffuse scleral thickening, choroidal folds, or a posterior “T-sign” is present. Inflammatory granuloma should remain in the differential when there are accompanying inflammatory findings or a compatible infectious or systemic inflammatory history.

FA and ICGA may be useful when vascular, melanocytic, or inflammatory alternatives remain plausible, but angiographic findings alone do not establish tumor histology (31). Concordant findings across OCT, FAF, angiography, ultrasonography, and serial photography increase clinical confidence and help identify progression. In a patient with known cancer, a compatible new or enlarging lesion should prompt systemic oncologic reassessment and ocular-oncology referral. In a patient without a known primary tumor, systemic imaging and oncology-directed evaluation should be guided by age, symptoms, lesion behavior, and overall pretest probability. Ocular biopsy is generally reserved for diagnostically uncertain cases in which tissue confirmation is expected to alter staging or management (32, 33).

#### 3.3.2 RPE-choroidal remodeling phenotype

Paraneoplastic choroidopathies usually present as diffuse or multifocal RPE-choroidal dysfunction rather than as a discrete mass. Suspicion increases when bilateral, rapidly progressive pigmentary abnormalities are accompanied by multifocal FAF changes, choroidal thickening, outer-retinal disruption, or serous retinal detachment and cannot be adequately explained by a more common retinal or inflammatory disorder (6, 34). The most clinically relevant clue is therefore not an isolated RPE abnormality but a bilateral and progressive multimodal pattern that is discordant with the expected course of central serous chorioretinopathy, inflammatory choroiditis, or age-related RPE disease.

In BDUMP, highly suggestive findings include multiple round or oval red patches at the level of the RPE with corresponding early hyperfluorescence on FA, a characteristic patchy or “giraffe-like” FAF pattern, and progressive outer-retinal or RPE disruption on OCT. Subretinal fluid, choroidal thickening, or diffuse melanocytic choroidal changes may provide additional support. The diagnostic contribution of imaging lies in recognition of the combined bilateral pattern and its progression over time, particularly when visual loss and structural abnormalities continue to worsen despite conventional retinal or anti-inflammatory treatment (35).

Important differential-diagnosis traps include central serous chorioretinopathy, VKH disease, posterior scleritis, inflammatory choroidopathy, and drug-related serous retinopathy. Central serous chorioretinopathy is more likely when a pachychoroid configuration and characteristic focal angiographic leakage are present without progressive multifocal RPE loss. VKH should be considered when multilobular serous detachments, choroidal folds, optic-disc leakage, inflammatory signs, or compatible systemic manifestations are present. Posterior scleritis may be suggested by ocular pain, scleral thickening, and ultrasonographic abnormalities. Treatment-related serous changes should be assessed according to the timing and class of systemic therapy.

FA and ICGA can define the distribution and level of retinal or choroidal vascular disturbance, while EDI-OCT or swept-source OCT can document choroidal thickness, outer-retinal integrity, RPE alteration, and subretinal fluid. Ultrasonography may be useful when diffuse choroidal thickening or posterior scleritis remains a consideration. Because no individual ocular test confirms a paraneoplastic mechanism, malignancy-directed evaluation should be based on the complete phenotype, patient age, systemic symptoms, cancer history, longitudinal progression, and the absence of a more plausible ocular explanation.

#### 3.3.3 Vitreoretinal infiltrative phenotype

The vitreoretinal infiltrative phenotype should be regarded as a trigger for a masquerade-syndrome work-up rather than as a diagnostic definition. Suspicion for vitreoretinal lymphoma increases in an older patient with persistent or recurrent intermediate or posterior uveitis, particularly when large vitreous cells form sheets, strands, or clumps; vitreous haze appears limited relative to the cellular burden; cystoid macular edema is absent or modest; or OCT demonstrates outer-retinal, subretinal, or sub-RPE hyperreflective deposits, RPE nodularity, or vertical hyperreflective lesions. Yellow-white retinal or subretinal lesions, granular hyperautofluorescent and hypoautofluorescent abnormalities, and recurrent lesions at different retinal locations may provide additional support. A transient or incomplete corticosteroid response does not exclude lymphoma (25, 36).

Widefield color photography and FAF are useful for identifying peripheral, multifocal, or clinically subtle lesions and for documenting their evolution. OCT should be performed through suspicious lesions whenever possible because localization to the outer retina, subretinal space, or sub-RPE compartment may help distinguish infiltrative disease from nonspecific vitreous inflammation. FA may demonstrate vascular leakage, staining, or areas of RPE disturbance, but angiographic findings are variable and should not be used independently to confirm or exclude lymphoma.

These findings must be interpreted against infectious and non-infectious mimickers. Viral retinitis should remain a priority when sharply demarcated necrotizing retinal lesions, prominent anterior uveitis, iris atrophy, hemorrhage, or occlusive arteritis are present. Toxoplasmic retinochoroiditis is considered when a focal necrotizing lesion is associated with a compatible chorioretinal scar. Syphilis, tuberculosis, sarcoidosis, intermediate or posterior uveitis, and selected white-dot syndromes should be evaluated according to the clinical context (37). These distinctions are probabilistic rather than exclusionary. When infectious retinitis remains plausible, aqueous or vitreous PCR testing for relevant pathogens should be performed rather than relying on the imaging phenotype alone.

When suspicion for vitreoretinal lymphoma remains high, diagnostic sampling should be coordinated in advance with the ocular pathology and molecular laboratory. If clinically safe, systemic corticosteroids should be withheld before diagnostic vitrectomy because prior treatment may reduce cellular yield. An undiluted vitreous specimen should be processed promptly for cytology, immunophenotyping or flow cytometry, IL-10 and IL-6 measurement, immunoglobulin gene-rearrangement analysis, and MYD88 L265P mutation testing. Targeted NGS may increase diagnostic confidence when sufficient material and local expertise are available, but should complement rather than replace cytopathologic assessment.

A negative individual test does not exclude lymphoma when the clinical phenotype and longitudinal course remain strongly suspicious. Discordant or repeatedly negative findings should prompt reconsideration of specimen quality, previous corticosteroid exposure, alternative sampling sites, and competing infectious or inflammatory diagnoses. Repeat sampling or biopsy of another involved site may occasionally be required. Brain MRI with contrast and appropriate hematology or neuro-oncology evaluation are necessary to assess associated central nervous system disease (25, 36).

#### 3.3.4 Diffuse neuroretinal dysfunction phenotype

When visual dysfunction is disproportionate to the funduscopic appearance, the diagnostic emphasis shifts from lesion morphology to functional localization. High-suspicion features include rapid or subacute bilateral visual deterioration, photopsia, nyctalopia, photosensitivity, ring or constricted visual-field defects, and relatively limited early funduscopic abnormalities. OCT may demonstrate attenuation of the ellipsoid zone, thinning of the outer nuclear layer, or more diffuse outer-retinal loss, while FAF may reveal a hyperautofluorescent transition ring or more widespread abnormalities corresponding to dysfunctional or degenerating retina (6, 26, 27).

Full-field ERG is central to objective functional characterization. Cancer-associated retinopathy may produce generalized rod-cone or cone-rod dysfunction, whereas melanoma-associated retinopathy is classically associated with an electronegative ERG caused by relatively preserved a-wave and reduced b-wave amplitudes, indicating predominantly bipolar-cell dysfunction. These findings are highly informative when consistent with the symptoms and cancer context but are not individually specific for a paraneoplastic mechanism. Visual-field testing can document the functional distribution and provide an objective measure for longitudinal monitoring.

Inherited retinal degeneration, toxic retinopathy, the AZOOR spectrum, non-paraneoplastic autoimmune retinopathy, and nutritional or metabolic retinal dysfunction should be considered. A longstanding or slowly progressive course, family history, or characteristic inherited phenotype favors retinal dystrophy. Medication exposure and the temporal relationship to treatment should be reviewed carefully because toxic retinopathy may produce overlapping outer-retinal and electrophysiologic abnormalities. AZOOR-spectrum disease may be more asymmetric or regionally distributed and should be interpreted in conjunction with FAF, OCT, and visual-field findings.

Antiretinal antibody testing may provide supportive evidence in a compatible clinical phenotype, but should not be treated as an isolated confirmatory test because antibody positivity is not disease-specific and assay results may vary between laboratories. ERG-confirmed dysfunction and a coherent clinical phenotype should generally precede interpretation of antibody results. Systemic evaluation should be directed by patient age, symptoms, cancer history, and the strength of the ocular phenotype rather than by antibody status alone. In a patient without a known malignancy, the scope of systemic investigation should be individualized and coordinated with the appropriate physician rather than based on a single positive antibody result (6).

#### 3.3.5 Treatment-imprint phenotype

Treatment-related posterior segment disease should be interpreted in relation to the class, timing, duration, dose, and sequence of systemic therapy. Findings that increase suspicion include a new abnormality arising after treatment initiation or dose modification, a bilateral or class-consistent pattern, progression during continued exposure, and improvement following treatment interruption or modification when clinically appropriate. A temporal relationship supports a treatment-related mechanism but does not prove causality, and deliberate rechallenge should not be undertaken solely to confirm an ocular adverse effect.

Different treatment classes may produce overlapping but recognizable patterns. MEK inhibitor-associated retinopathy may present with bilateral, multifocal, shallow subretinal fluid and limited or absent corresponding leakage on FA. Immune checkpoint inhibitors may produce anterior or posterior uveitis, retinal vasculitis, optic-disc leakage, choroidal thickening, or a VKH-like phenotype with serous retinal detachment. Radiation retinopathy typically manifests as a delayed microangiopathy with hemorrhages, cotton-wool spots, macular edema, capillary nonperfusion, or neovascular complications. Chemotherapy and other targeted therapies may additionally produce vascular events, macular edema, or toxic outer-retinal injury (28, 38).

The main diagnostic traps are tumor progression, opportunistic infection, pre-existing diabetic or hypertensive retinopathy, unrelated retinal vascular occlusion, central serous chorioretinopathy, and non-treatment-related inflammatory disease. Opportunistic viral retinitis should be considered particularly in immunocompromised patients with necrotizing lesions or occlusive vasculitis. New choroidal or retinal lesions in a patient receiving anticancer therapy should not automatically be attributed to toxicity because metastatic or infiltrative progression may occur during treatment.

OCT can quantify retinal thickness, subretinal or intraretinal fluid, and outer-retinal integrity, while OCTA can document changes in retinal and choroidal microvasculature. FA is appropriate when leakage, vasculitis, capillary nonperfusion, or neovascularization is suspected, and ICGA may be required when choroidal inflammation or perfusion abnormalities remain uncertain. Widefield imaging can document peripheral vascular or inflammatory involvement and provide a baseline for longitudinal comparison.

Decisions regarding continuation, interruption, or modification of anticancer treatment should be made through multidisciplinary discussion. Management should reflect the severity and reversibility of the ocular event, the strength of the causal association, the risk of permanent visual loss, availability of local ophthalmic treatment, and the systemic oncologic benefit of continuing therapy. Ophthalmic findings should therefore be communicated using objective measures and documented longitudinal change rather than a nonspecific attribution of “drug toxicity.”

Across all five phenotypes, prompt escalation should be considered when imaging demonstrates a new or enlarging choroidal mass, bilateral or multifocal unexplained RPE-choroidal change, persistent or corticosteroid-resistant vitreoretinal inflammation, rapid visual dysfunction disproportionate to the fundus appearance, or a severe new abnormality temporally related to anticancer treatment. Multimodal imaging should define the dominant site of involvement, characterize biological behavior, identify discordant findings, and document evolution over time. Its principal actionable role is to determine whether observation, additional ocular testing, infectious or inflammatory investigation, tissue or fluid sampling, systemic imaging, oncology referral, or treatment-toxicity review is warranted. The recommended escalation pathway is summarized in **Table 3**.

**Table 3:** Practical framework for translating multimodal posterior segment imaging into oncologic clinical reasoning.

Suspicious phenotype	Features increasing suspicion	Priority mimickers to exclude	Additional tests and highest-confidence diagnostic step
Exudative choroidal mass	New or enlarging creamy-yellow or amelanotic lesion, multifocality, subretinal fluid, altered choroidal contour	Amelanotic melanoma, hemangioma, inflammatory granuloma, posterior scleritis	EDI/SS-OCT, FAF, standardized A/B-scan ultrasonography; FA/ICGA when vascular or inflammatory differentiation remains uncertain; systemic staging and ocular-oncology referral; biopsy when diagnostic uncertainty remains and tissue confirmation would alter management
RPE-choroidal remodeling	Bilateral multifocal RPE disturbance, rapidly evolving FAF abnormality, choroidal thickening, unexplained serous detachment	Central serous chorioretinopathy, VKH disease, posterior scleritis, inflammatory choroidopathy, drug-related change	Bilateral EDI/SS-OCT, FAF, FA/ICGA, ultrasonography when appropriate, and ERG if diffuse dysfunction is present; malignancy-directed systemic assessment when the phenotype is progressive and otherwise unexplained
Vitreoretinal infiltrative	Persistent or recurrent vitritis, cells in sheets or clumps, limited haze relative to cellular burden, retinal/subretinal/sub-RPE deposits, absent or limited macular edema, transient or incomplete corticosteroid response	Viral or toxoplasmic retinitis, syphilis, tuberculosis, sarcoidosis, intermediate/posterior uveitis, selected white-dot syndromes	UWF photography, FAF, OCT, FA ± ICGA; aqueous/vitreous infectious PCR when indicated; coordinated diagnostic vitrectomy for cytology, flow cytometry, IL-10/IL-6, IGH/IGK clonality, and MYD88 analysis; targeted NGS as an adjunct; brain MRI and hematology/neuro-oncology assessment
Diffuse neuroretinal dysfunction	Photopsia, nyctalopia, or field loss disproportionate to fundus appearance, bilateral outer-retinal disruption, abnormal ERG	Inherited retinal degeneration, toxic retinopathy, AZOOR spectrum, non-paraneoplastic autoimmune retinopathy	Full-field ERG, visual fields, OCT, FAF, and medication review; systemic cancer evaluation guided by the complete phenotype; antiretinal antibodies as supportive rather than standalone evidence
Treatment-imprint	New abnormality temporally related to systemic therapy, bilateral or treatment-class-consistent pattern, improvement after modification when clinically appropriate	Tumor progression, opportunistic infection, unrelated uveitis, diabetic/hypertensive disease, retinal vascular occlusion	Treatment timeline, OCT/OCTA, FA ± ICGA, and targeted systemic vascular or infectious evaluation; multidisciplinary assessment before systemic therapy is modified

AZOOR, acute zonal occult outer retinopathy; EDI, enhanced depth imaging; ERG, electroretinography; FA, fluorescein angiography; FAF, fundus autofluorescence; ICGA, indocyanine green angiography; IGH, immunoglobulin heavy chain; IGK, immunoglobulin kappa light chain; MRI, magnetic resonance imaging; NGS, next-generation sequencing; OCT, optical coherence tomography; OCTA, optical coherence tomography angiography; PCR, polymerase chain reaction; RPE, retinal pigment epithelium; SS, swept-source; UWF, ultra-widefield; VKH, Vogt-Koyanagi-Harada disease.

Several findings should lower the threshold for escalation: a new choroidal mass in an older patient; bilateral, multifocal, or peripheral abnormalities not explained by a common retinal disorder; recurrent or atypical inflammation; rapid visual deterioration disproportionate to fundus findings; progression despite conventional ophthalmic treatment; and a new posterior segment abnormality during anticancer therapy. None is independently diagnostic. Their value lies in prompting timely exclusion of mimickers and selection of the most appropriate confirmatory or multidisciplinary next step.

### 3.4 AI-assisted surveillance: current evidence and limitations

Artificial intelligence should currently be regarded as a research tool rather than an independent diagnostic system for malignancy-related retinal and choroidal disease. The available evidence is task- and modality-specific. Interpretable multimodal models have demonstrated that choroidal tumor categories can be distinguished while retaining clinically understandable feature-level outputs (39). These studies provide proof of concept for rare ocular tumors, but they do not validate AI diagnosis of vitreoretinal lymphoma, paraneoplastic retinopathy, or treatment-related retinal toxicity. The most plausible near-term role for AI in the disorders reviewed here is longitudinal comparison rather than single-image classification. Potential quantitative targets include lesion dimensions, subretinal fluid, RPE disruption, choroidal thickness, vitreous haze, vascular abnormality, and outer-retinal integrity (40). Their clinical meaning depends on change over time, including new onset, progression, recurrence, regression after systemic treatment, or deterioration after anticancer therapy exposure (41). Image registration, lesion segmentation, and standardized change detection may reduce variability in serial assessment and facilitate communication between ophthalmologists and oncologists (42, 43). Future foundation or multimodal models may also support comparison across imaging modalities, but their outputs must remain linked to clinically interpretable features and a defined decision context (9, 44, 45).

The limitations are particularly important in rare onco-ophthalmic disease. Widefield color photography may document lesion distribution but can provide an incomplete signal when the relevant abnormality is vitreous, intraretinal, subretinal, or sub-RPE, or when media haze obscures the fundus. A normal or nonspecific photographic appearance should therefore not be used to exclude an infiltrative disorder. Current ophthalmic AI evidence remains substantially stronger in high-volume diseases such as diabetic retinopathy and age-related macular degeneration (46). Malignancy-related datasets are smaller and more heterogeneous, with variable imaging protocols, substantial class imbalance, and limited reference labels anchored in cytology, molecular testing, histopathology, or well-defined oncologic outcomes (47–49).

Future systems will require multicenter datasets, harmonized acquisition protocols, prespecified clinical tasks, molecularly or pathologically anchored ground truth where available, external validation, transparent error analysis, and human-in-the-loop assessment before clinical implementation. Until such evidence is available, AI should be presented as a potential aid for serial comparison, lesion tracking, and treatment-toxicity surveillance. Final interpretation must remain based on expert clinical judgment, multimodal concordance, exclusion of relevant mimickers, and systemic oncologic context.

## 4 Discussion

This narrative review proposes that retinal and choroidal abnormalities associated with systemic malignancy should be interpreted as suspicion-triggering imaging patterns with potential biomarker relevance, rather than as validated biomarkers or independently diagnostic signs. Their clinical value lies in their ability to reflect a probable oncologic mechanism, prioritize relevant differential diagnoses, and prompt an appropriate confirmatory investigation. Posterior segment abnormalities may result from metastatic dissemination, paraneoplastic immune-mediated injury, hematologic or lymphomatous infiltration, or anticancer treatment-related toxicity. This approach is consistent with the broader evolution of oncologic imaging toward biologically informed, longitudinal, and treatment-oriented interpretation rather than lesion detection alone (50, 51). Nevertheless, imaging findings should not be separated from patient age, systemic cancer history, treatment exposure, immune status, disease tempo, and response to previous therapy.

The principal contribution of this review is therefore not the description of individual malignancy-related ocular disorders, which has been addressed in disease-specific reviews, but their organization into a shared phenotype-to-confirmation framework (3, 6, 25, 28, 32, 34, 36, 38). Unlike a conventional disease-centered ocular oncology overview, the proposed framework begins with the abnormal posterior segment pattern encountered by the clinician and asks five practical questions: which anatomical compartment is predominantly involved, what is the dominant morphology, which oncologic mechanism is plausible, which competing diagnoses must first be excluded, and which additional investigation offers the highest diagnostic value. The clinical endpoint is not an image-based malignant diagnosis, but timely escalation from an atypical ocular finding to the most appropriate ocular, molecular, laboratory, or systemic evaluation.

Multimodal imaging is most useful when each modality is selected to address a specific unresolved diagnostic question. OCT localizes abnormalities to the choroid, RPE, sub-RPE space, outer retina, or vitreoretinal interface and demonstrates associated exudation or tissue infiltration. Fundus autofluorescence identifies the distribution of secondary RPE stress, whereas widefield imaging may reveal peripheral or multifocal disease that is underestimated on conventional examination (8, 9). Fluorescein and indocyanine green angiography provide complementary information regarding retinal and choroidal vascular behavior, and standardized ultrasonography is particularly relevant when a solid choroidal lesion or posterior scleritis remains in the differential diagnosis. For example, an exudative choroidal mass becomes more suspicious for metastasis when an amelanotic or creamy-yellow lesion is accompanied by subretinal fluid, an irregular choroidal contour, multifocality, or documented progression. However, differentiation from amelanotic melanoma, choroidal hemangioma, inflammatory granuloma, and posterior scleritis may require FA, ICGA, A- and B-scan ultrasonography, systemic staging, and, in selected indeterminate cases, tissue diagnosis (31–33).

The vitreoretinal infiltrative phenotype most clearly demonstrates why imaging should trigger, rather than conclude, the diagnostic process. Suspicion for vitreoretinal lymphoma increases in an older patient with persistent or recurrent intermediate or posterior uveitis, particularly when large vitreous cells form sheets or clumps, vitreous haze is limited relative to the cellular burden, cystoid macular edema is absent or modest, or OCT demonstrates outer-retinal, subretinal, or sub-RPE hyperreflective deposits. A transient or incomplete corticosteroid response does not exclude lymphoma. Conversely, sharply demarcated necrotizing retinal lesions, prominent anterior uveitis, iris atrophy, or occlusive arteritis may increase suspicion for viral retinitis, while a focal necrotizing lesion adjacent to a compatible chorioretinal scar may support toxoplasmic retinochoroiditis. Syphilis should likewise remain in the differential diagnosis of atypical panuveitis or retinal-detachment presentations (37). These distinctions remain probabilistic, and infectious retinitis should be excluded by pathogen-directed aqueous or vitreous PCR when clinically plausible.

When suspicion for vitreoretinal lymphoma persists, diagnostic sampling should be coordinated with the ocular pathology and molecular laboratory. Cytology remains central, but diagnostic confidence may be increased by combining cytologic assessment with immunophenotyping or flow cytometry, IL-10 and IL-6 measurement, immunoglobulin gene rearrangement analysis, and MYD88 mutation testing. Targeted next-generation sequencing may provide additional information when adequate material and expertise are available, but should be interpreted as complementary to, rather than a replacement for, cytopathologic assessment. Importantly, a negative individual test does not exclude lymphoma when the phenotype and clinical course remain strongly suspicious. Brain MRI and hematology or neuro-oncology assessment are also required to evaluate associated central nervous system disease (25, 36).

The other proposed phenotypes similarly require confirmation beyond routine imaging. In diffuse neuroretinal dysfunction, photopsia, nyctalopia, visual field loss, and outer retinal disruption that appear disproportionate to the fundus findings should prompt electroretinography and systemic correlation. Antiretinal antibody testing may provide supportive evidence, but should not be interpreted in isolation because antibody positivity is not specific for paraneoplastic disease (6, 26, 27). Likewise, a treatment-imprint phenotype should not be diagnosed solely on the basis of temporal association. Tumor progression, opportunistic infection, unrelated inflammatory disease, vascular occlusion, and common retinal comorbidities should be considered before attributing a new finding to anticancer therapy or recommending treatment modification. Decisions regarding interruption or modification of systemic treatment should therefore be made jointly by the ophthalmologist and treating oncologist (1, 28, 38). More broadly, the tumor microenvironment is a dynamic determinant of treatment response and resistance, reinforcing the need to interpret ocular changes in relation to systemic disease behavior and the therapeutic context (52).

The clinical relevance of many malignancy-related posterior segment findings is longitudinal rather than static. Serial imaging may establish whether a lesion is stable, progressive, recurrent, regressive following systemic treatment, or temporally associated with a particular anticancer therapy. **Table 3** translates this principle into a phenotype-specific diagnostic framework. In this setting, artificial intelligence should be considered a potential supportive layer for image registration, serial comparison, lesion or fluid quantification, and treatment-toxicity surveillance rather than an autonomous diagnostic system. Recent work on interpretable multimodal models for choroidal neoplasia demonstrates the feasibility of feature-level AI reasoning, but these results cannot be assumed to generalize directly to systemic malignancy-related posterior segment disease (39, 41).

The rarity and heterogeneity of these disorders create substantial obstacles to AI development, including small and imbalanced datasets, inconsistent diagnostic reference standards, variable imaging protocols, missing imaging modalities, device-related domain shift, and limited external validation. A model trained using widefield color photography, for example, cannot be assumed to retain equivalent performance when applied to autofluorescence images, images obtained on a different device, or a clinically distinct disease population. Future studies should therefore determine the incremental value of individual and combined modalities, rather than assuming that multimodal input necessarily improves diagnostic performance. Multicenter datasets, standardized acquisition protocols, harmonized outcome definitions, external validation, transparent model outputs, and human-in-the-loop oversight will be required before clinical implementation can be considered (9, 46–51).

This review has several limitations. Its narrative design did not include formal risk-of-bias assessment, quantitative evidence grading, or pooled estimates of diagnostic accuracy. Much of the available evidence for rare malignancy-related ocular manifestations is derived from retrospective series, small cohorts, and diagnostically informative case reports, creating susceptibility to selection and publication bias. Imaging protocols, diagnostic confirmation methods, and follow-up intervals were heterogeneous across publications. In addition, the five phenotypes were developed as an interpretive clinical model and have not been prospectively validated. Overlap between phenotypes is expected, particularly in patients with simultaneous tumor-related, immune-mediated, vascular, and treatment-related abnormalities. Consequently, the framework should be regarded as a structured aid to clinical suspicion and diagnostic escalation rather than as a classification system with established sensitivity, specificity, or predictive value.

Despite these limitations, a phenotype-to-confirmation approach may help clinicians recognize when a posterior segment abnormality does not follow the expected behavior of a common retinal or inflammatory disorder. Its principal value lies in maintaining an appropriately high index of suspicion while preserving diagnostic uncertainty, explicitly considering relevant mimickers, and linking multimodal findings to the next clinically justified investigation. Prospective multicenter studies will be required to determine the reproducibility, diagnostic performance, and clinical utility of the proposed phenotypes.

### 4.1 Limitations and future directions

Several limitations should be considered when interpreting the findings of this review. First, the review employed a narrative rather than a systematic design. Although a narrative approach was appropriate for a heterogeneous topic encompassing rare metastatic, paraneoplastic, hematologic, lymphomatous, infiltrative, and treatment-related posterior segment disorders, it does not ensure exhaustive evidence retrieval and does not permit formal risk-of-bias assessment, quantitative evidence grading, or pooled data synthesis. A targeted search strategy, explicit eligibility rationale, and predefined qualitative synthesis approach were used to improve transparency; nevertheless, selection and publication bias cannot be excluded. Second, the available evidence is heterogeneous with respect to disease entities, patient populations, imaging modalities, acquisition protocols, diagnostic reference standards, follow-up duration, outcome measures, and study design. Many malignancy-related posterior segment manifestations have been described primarily in case reports, small case series, retrospective cohorts, and expert reviews. Moreover, diagnostically striking or molecularly confirmed cases may be more likely to be published than atypical, unconfirmed, or negative cases. This may overestimate the apparent specificity of individual imaging patterns and limits their generalizability to routine clinical populations. Third, diagnostic confirmation was inconsistent across the included literature. Depending on the disease entity, confirmation was based on systemic oncologic findings, treatment response, histopathology, cytology, immunophenotyping, molecular testing, laboratory findings, or longitudinal clinical behavior. Consequently, apparently similar imaging abnormalities may have been classified using different reference standards. The five proposed phenotypes may also overlap within the same patient, particularly when metastatic, immune-mediated, vascular, infectious, and treatment-related mechanisms coexist. The framework should therefore be regarded as a clinically orienting model for diagnostic escalation rather than as a mutually exclusive classification or validated scoring system. Fourth, the present review does not provide pooled estimates of sensitivity, specificity, predictive value, or likelihood ratios for individual imaging signs or phenotype combinations. Most available studies were not designed as diagnostic accuracy studies and did not include sufficiently representative control groups or clinically relevant mimickers. Future validation studies should therefore include both confirmed malignancy-related cases and non-malignant inflammatory, infectious, vascular, degenerative, and primary ocular neoplastic mimickers. In addition to diagnostic accuracy, such studies should assess interobserver reproducibility, calibration, incremental value beyond conventional clinical information, and whether use of the framework meaningfully changes diagnostic timing or patient management. Fifth, differences in imaging devices, acquisition protocols, image quality, field of view, follow-up intervals, and availability of individual modalities limit direct comparison across studies. Future research should establish minimum multimodal imaging datasets and standardized reporting criteria for each phenotype. Device metadata, treatment timing, systemic disease status, and longitudinal changes should be recorded consistently to support cross-center comparison and future quantitative analysis.

AI-assisted surveillance in onco-ophthalmology remains conceptual. Malignancy-related posterior segment disorders are rare and heterogeneous and are insufficiently represented in standardized ophthalmic datasets. Potential barriers include small and imbalanced samples, uncertain or inconsistent reference standards, missing imaging modalities, device-related domain shift, spectrum bias, and inadequate external validation. Accordingly, future AI systems should not be developed exclusively from highly selected, confirmed cases. Training and validation datasets should include relevant mimickers, negative examinations, incomplete multimodal studies, and images acquired using different devices and clinical workflows.

Future research should prioritize multicenter onco-ophthalmic registries linking ocular findings with cancer type, systemic disease status, anticancer treatment exposure and timing, ocular and systemic treatment response, and histologic, cytologic, molecular, laboratory, or longitudinal confirmation when available. Such registries would permit prospective or multicenter retrospective evaluation of the proposed phenotypes, identification of the most informative modality combinations, and development of standardized reporting systems. The principal role of future AI tools should be to support image registration, serial comparison, quantitative change detection, risk flagging, and multidisciplinary communication rather than autonomous diagnosis. Any clinical implementation would require transparent outputs, bias assessment, external validation, prospective evaluation, and human-in-the-loop oversight. The principal limitations of the current evidence base and corresponding research priorities are summarized in **Table 4**.

**Table 4:** Limitations of the current evidence and future research priorities.

Current limitation	Clinical or research implication	Future research priority
Narrative review design without formal risk-of-bias assessment or quantitative evidence grading	Limits exhaustive evidence synthesis and increases susceptibility to selection and publication bias	Conduct disease-specific systematic reviews and prospective evidence synthesis when sufficiently comparable data become available
Rare and heterogeneous disease entities supported predominantly by case reports, small series, and retrospective cohorts	Limits generalizability and may overrepresent diagnostically striking or confirmed presentations	Establish multicenter onco-ophthalmic registries containing representative cases, mimickers, and negative examinations
Variable or incomplete diagnostic confirmation	Reduces certainty that an imaging pattern is directly related to malignancy or its treatment	Apply predefined reference standards incorporating histologic, cytologic, molecular, laboratory, systemic oncologic, and longitudinal confirmation as appropriate
Lack of diagnostic accuracy data and representative comparison groups	Sensitivity, specificity, predictive value, likelihood ratios, and incremental clinical value remain unknown	Validate the phenotypes against infectious, inflammatory, vascular, degenerative, and primary ocular neoplastic mimickers
Proposed phenotypes are overlapping and have not been externally validated	The framework cannot currently be used as a diagnostic classification or scoring system	Assess interobserver agreement, reproducibility, calibration, diagnostic performance, and effect on clinical decision-making
Variable imaging devices, protocols, image quality, and follow-up intervals	Limits cross-study comparison, quantitative synthesis, and longitudinal assessment	Define minimum multimodal imaging datasets, standardized acquisition protocols, device metadata, and uniform follow-up time points
Limited linkage between imaging findings and systemic cancer status or treatment response	Restricts assessment of oncologic relevance and prognostic value	Link imaging trajectories with cancer type, systemic disease status, treatment exposure, treatment response, and ocular outcomes
Small, imbalanced, and non-standardized datasets for AI development	Increases the risks of overfitting, spectrum bias, domain shift, and poor external generalizability	Develop interpretable human-in-the-loop models using multicenter datasets, relevant mimickers, external validation, bias assessment, and prospective workflow evaluation

AI, artificial intelligence; OCT, optical coherence tomography.

## 5 Conclusions

This narrative review proposes a phenotype-to-confirmation framework comprising five suspicion-triggering imaging phenotypes: exudative choroidal mass, RPE–choroidal remodeling, vitreoretinal infiltrative, diffuse neuroretinal dysfunction, and treatment-imprint phenotypes. By linking dominant imaging patterns with plausible oncologic mechanisms, the framework may help clinicians recognize atypical presentations, prioritize relevant mimickers, and select appropriate confirmatory investigations. These phenotypes are not validated biomarkers or definitive diagnoses; their significance depends on systemic cancer history, treatment exposure, immune status, disease tempo, and longitudinal evolution. Ophthalmic imaging should therefore be regarded as the beginning of the diagnostic pathway, prompting targeted multimodal imaging, laboratory or molecular testing, ocular sampling, or systemic assessment when appropriate. Future multicenter studies should evaluate the reproducibility, diagnostic performance, and clinical utility of the proposed framework. AI may support longitudinal monitoring and multidisciplinary communication, but should remain interpretable, externally validated, human-supervised, and complementary to expert clinical judgment.
